# Human development, greenhouse gas emissions and sub-national mitigation burdens: a Brazilian perspective

**DOI:** 10.1007/s43621-021-00044-9

**Published:** 2021-08-09

**Authors:** Geanderson Ambrósio, Dênis Antônio Da Cunha, Marcel Viana Pires, Luis Costa, Raiza Moniz Faria, Angelo Costa Gurgel

**Affiliations:** 1 Universidade Federal de Viçosa, Viçosa, Minas Gerais Brazil; 2 Potsdam Institute for Climate Impact Research, Potsdam, Germany; 3 São Paulo School of Economics, Fundação Getúlio Vargas, São Paulo, Brazil

## Abstract

International frameworks for greenhouse gas (GHG) mitigation usually disregard country-specific inequalities for the allocation of mitigation burdens. This may hinder low developed regions in a country from achieving development in a socioeconomic perspective, such as the Sustainable Development Goals (SDGs) of eradicating poverty (SDG1) and hunger (SDG2). We use observed data (1991–2010) of carbon dioxide equivalent (CO_2_eq) emissions and a sub-national human development index (MicroHDI, range [0, 1]) for Brazilian microregions to design a framework where regional mitigation burdens are proportional to the MicroHDI, without compromising national mitigation pledges. According to our results, the less developed Brazilian regions have not been basing their development in emission-intensive activities; instead, the most developed regions have. Between 2011 and 2050, Brazilian cumulative emissions from the sectors most correlated with MicroHDI are expected to be 325 Gt CO_2_eq, of which only 50 Gt are associated with regions of MicroHDI < 0.8. Assuming a national GHG mitigation target of 56.5% in 2050 over 2010 (consistent with limiting global warming to 2 ºC), Brazil would emit 190 Gt CO_2_eq instead of 325 Gt and the 135 Gt reduction is only accounted for by regions after reaching MicroHDI ≥ 0.8. Allocating environmental restrictions to the high-developed regions leaves ground for the least developed ones to pursue development with fewer restrictions. Our heterogeneous framework represents a fairer allocation of mitigation burdens which could be implemented under the concepts of green economy. This work could be an international reference for addressing both environmental and socioeconomic development in developing countries at sub-national level as emphasized by the SDGs.

**Supplementary Information:**

The online version contains supplementary material available at 10.1007/s43621-021-00044-9.

## Introduction

Human demands have been overloading the biosphere’s resilience with greenhouse gases (GHG) emissions over the recent decades [1, 2]. This overload makes it difficult to determine the point at which the expansion of economic systems will exceed sustainable levels supported by the biosphere [3, 4]. Moreover, the additional cost of this growth may exceed the flow of additional welfare benefits [5, 6] especially for the poorest if an unfair GHG mitigation burden is allocated to regions with low access to other basic needs, such as health, wealth and education. Associated with the unchecked release of GHG comes an increased incidence of extreme and adverse weather conditions such as prolonged droughts and floods—whose negative effects in turn are expected to affect especially the poorest and most vulnerable regions [1].

Responsible production and consumption are key factors to achieve local and global sustainable development in diverse aspects, including, but not limited to, economics, ecology, and social sciences [7]. A broad concept of development is accounted by the 17 United Nations Sustainable Development Goals (SDGs), which emphasize the importance of human development in social and economic aspects in addition to environmental concerns and explicitly define targets for it [8]. While SDGs define a comprehensive set of targets, other approaches build upon pathways to achieve them. The implementation of green economy principles enable designing pathways to ensure sustainability in the development process [9]. According to the report “Towards a Green Economy: Pathways to Sustainable Development and Poverty Eradication” from United Nations Environment Programme—UNEP [10], green economy is an important tool for sustainable development; one that is inclusive and can drive economic growth, employment, and poverty eradication, whilst maintaining the healthy functioning of the Earth’s ecosystems”. Green Economy initiatives allows for “low-carbon, resource efficient and socially inclusive” countries to grow, “while significantly reducing environmental risks and ecological scarcities” [10]. In this sense, Green Economy is closely related to SDGs and can be taken as framework to develop potential pathways to achieve multiple sets of SDGs [11].

It is of crucial importance to account for these frameworks to understand under what circumstances socioeconomic human development takes place while reducing GHG emission [12–14] and how to allocate mitigation burdens without constraining the other aspects of development, especially of the least developed regions. Some GHG emission sources closely relate to production patterns and economic development, such as fossil fuel combustion [12], but others barely relate to it, such as deforestation. In order to establish fair GHG emission reduction burdens, it is necessary to consider emission sources most associated with human development. Accounting for the public characteristics of the externalities of environmental degradation and the need to reduce GHG emissions [13], mitigation commitments are expected to be undertaken among developed and developing societies according to their differentiated capacities [1, 14]. Moreover, production patterns are ultimately defined by consumption, which is much associated with high levels of well-being.

Some methodological approaches have emerged to respond to the pressure of considering mitigation and human development jointly. Although the calculation of historical contributions to global warming have some misspecifications [2, 15], the recognition that different societies have different responsibilities regarding global warming remains valid [14]. This recognition enhances frameworks to take past development into account when defining mitigation burdens of least developed regions to achieve country or global mitigation targets [12]. The importance of these approaches rely on providing the opportunity to reduce the development gap between societies while still being able to achieve GHG mitigation pledges and is aligned with green economy as it provides opportunities to reduce the development gap between societies while still being able to achieve global and local GHG mitigation pledges [16, 17].

In order to consider human development as a parameter to mitigation commitments, Costa and co-workers [12] developed a country level emission estimation framework that allocates CO_2_ reductions from fossil fuel combustion based on the Human Development Index (HDI) [18]. The authors used HDI as a summary measure reflecting the achievement of a country in three basic dimensions of development: a long healthy life, access to knowledge, and decent living standards. They demonstrate a positive correlation between HDI and CO_2_ emissions from fossil fuel combustion in different countries and assume that development and emission trends unfold as in the past, following a so-called Development As Usual (DAU) trajectory. They propose a framework where only developed countries should reduce emissions, enabling developing ones to maintain development based on more emission intensive activities without compromising global mitigation targets if they prefer or require such activities. Their framework enables reducing development gaps between countries and describes pathways for developing ones to achieve a larger set of SDGs with less restrictions from the environmental perspective, if they want to do so.

Costa and co-workers [12] presented this framework at the country level as each country presents specific challenges to implement sustainable development [19]. Their approach, however, fails to capture sub-national dynamics and heterogeneities, which are especially relevant for developing countries [20, 21]. Sub-national development pathways have also global implications because the most of the world’s population lives in developing countries, where it is even more important to have fair development standards, both within the country and compared to other countries. For a continent-sized nation as Brazil, this is especially important, but such application has not yet been provided. In order to advise on the allocation of GHG mitigation burdens, developing countries require comprehensive studies from the socioeconomic point of view, as to ensure that stipulated reduction targets do not jeopardize the achievement of better living standards [22, 23]. Developing countries like Brazil usually base their development in agricultural activities, therefore, it requires broadening the scope of emissions sources to other human development related emissions besides fossil fuel combustion. Therefore, an inside country approach that captures Brazilian heterogeneity is required, because the country’s diverseness would not be evident if Brazil were treated as a single observation in a sample. Moreover, it has to account for specific notional policies, such as the Brazilian National Police on Climate Change [24] and its Nationally Determined Contribution (NDC) [25], as they play a major role in the national mitigation pledges.

Our study addresses this context by proposing a sub-national framework for the allocation of mitigation burdens from GHG emission sources closely correlated to a Brazilian internal measure of human development. We then apply it to Brazilian intern regions in a way that mitigation efforts of each region are commensurate with its standards of living without compromising the Brazilian national mitigation targets. The framework operates by allowing emissions to grow in each region until a minimum standard of wellbeing is achieved while developed regions are responsible for a major part of the national mitigation effort. Taking subnational heterogeneities into account while addressing human development and CO2 mitigation is in line with (i) the Common But Differentiated Responsibility (CBDR) principle of the 1992 United Nations Framework Convention on Climate Change (UNFCCC) [12, 17], (ii) the concepts of Green Economy [9, 10] and to (iii) SDGs associated with Climate Action (SDG13), No Poverty (SDG1), Good Health and Well-Being (SDG3), Quality Education (SDG4) and Decent Work and Economic Growth (SDG8) [8].

Our main objective is to present a more equally distributed mitigation reduction scheme inside Brazil and discuss its implications. More specifically, we intend to: (i) develop an index that measures development at a Brazilian regional level; (ii) project the index between 1991 and 2050; (iii) identify the correlation between the projected index and observed GHG emissions; (iv) extrapolate their correlation in order to project GHG emissions between 1991 and 2050; (v) propose an emission allocation framework which considers standards of living (by using the developed index) when assigning regional GHG mitigation burdens to achieve a national reduction target and (vi) analyze the results of the proposed framework.

The following section describes the analytical framework. Section Results and Discussion regards to three different issues: (i) developing and projecting an index that measures development at Brazilian regions; (ii) projecting GHG emissions; and (iii) developing the mitigation scheme that considers the standards of living of Brazil’s regions when allocating mitigation burdens. In the last part of the results section, we compare our reduction framework to other possible allocation scenarios of emission reduction burdens. We also demonstrate how to balance economic development with environmental responsibility, extending prior cross-country modeling to apply within Brazil, where different regions are developing at different rates. The final section presents our conclusions.

## Analytical framework

### Data

We used the 587 Brazilian *microregions* as the research unit. A *microregion* is a legal geographical division without a central political unit, which compresses groups of municipalities with common social, agricultural, industrial, and trade characteristics [26]. In the present research they act similarly to the minimum comparable area [27] because their implementation allows a historical series study of a given geographical area. This is possible even if the area of some municipalities have changed, because the area of the *microregions* have not changed in the period analyzed. As the features and interests of *microregions* are mostly related, they may take joint actions in order to be stronger in their demands over the state and federal governments.

As source for GHG emission data, we used gridded information on GHG emissions obtained from the Emission Database for Global Atmospheric Research (EDGAR) v4.2 FT2010 [28], which provides global data in 0.1º × 0.1º grid. Even though the database presents country-level emissions lower than baseline, it is consistent within each country, therefore being suitable for this sub-national application. The GHG emissions were quantified as carbon dioxide equivalent (CO_2_eq) using AR5’s Global Warming Potential for 100 years for methane (CH_4_), nitrous oxide (N_2_O) and carbon dioxide (CO_2_) [29]. We used emission from energy, industrial processes, residues and agriculture sectors, which are available from 2000 to 2010 (the further disaggregation of emission sources to compose these sectors is available at [28]). Although emissions from deforestation are huge in Brazil [30], this sector historically does not correlate with human development measured at state level [31]: −0.2 in 1991, −0.15 in 2000 and −0.31 in 2010 (Supplementary File 1). On the other hand, the sectors considered in this research present correlation between 0.28 and 0.56, at state level, for the same years (Supplementary File 1). By choosing these sectors, we intend to broaden the research by covering more human development related sectors to account for the Brazilian heterogeneity without getting into the only sector that does not correlate to it.

In order to elaborate an index measuring human development at the *microregional* level, we used the data of the Municipal Human Development Index (MHDI) [31] (Supplementary material), which is developed by United Nations and is available for Brazilian municipalities for the years 1991, 2000 and 2010. MHDI takes information on health, education and wealth at the city level directly from the Brazilian Census, similarly to the process employed to develop the country based HDI [18]. Just as HDI, MHDI receives criticism for treating income, health and education as substitutes. However, both have been used to compare the social and economic development within and among societies over time [32–34]. Furthermore, projecting HDI plays an important role in raising the policy profile for health and education once their predictability is reasonably good at the country level [35]. In the next section, we describe the process to convert the MHDI from the city level to an index suitable to our research, the *microregion*. The analysis was performed with Stata 12.

### Generating and extrapolating the *Microregional* Human Development Index

We balanced the MHDI by the population from each municipality in a *microregion* with its total population. This way, we transformed MHDI in order to reflect the general human development experienced by the whole population in each *microregion*. Thus, we got a close estimate to the human development level that the population at each *microregion* experiences. We named this indicator as Brazilian *Microregional* Human Development Index (MicroHDI).

In Eq. 1, the MicroHDI ($d_{i,t}$) for the *microregion i* $(i = 1,...,\;558)$ in time *t* $\left( {t = 1991,\;\,2000,\;\,2010} \right)$ was determined as the MHDI balanced by the population of municipality $(m)$ belonging to the *microregion* $(i)$; $MHDI_{m,t}$ is the MHDI of municipality *m* $(m = 1,...,\;n)$ in time *t* and $Pop_{m,t}$ is the total population. Data used for Eq. 1 is available in Supplementary File 2

1 $${d_{i,t} = \frac{{\sum\nolimits_{{{\text{m}} = {1}}}^{{\text{n}}} {\left[ {MHDI_{m,t} \left( {Pop_{m,t} } \right)} \right]} }}{{\sum\nolimits_{{{\text{m}} = {1}}}^{{\text{n}}} {Pop_{m,t} } }}}\;\forall \;m \in i$$

We applied the logistic function to project estimations of the MicroHDI from 1991 to 2050. Future increments in human development slowdown in time for highly developed economies [18, 36], which is simulated by the sigmoidal shape of the logistic function. E﻿q. 2 estimates the expected MicroHDI $({\tilde{d}_{i,t} })$ of each *microregion* ranging from 0 to 1, in which the coefficient $a_{i}$ represents how fast a *microregion* develops, $b_{i}$ is the independent adjustment term and $t$ is time.

2 $${\tilde{d}_{i,t} = \frac{{1}}{{{1} + e^{{ - a_{i} t + b_{i} }} }}}$$

After fitting each *microregion*’s MicroHDI to the curve using observed data for the years 1991, 2000 and 2010, the estimated coefficients ($a_{i}$ and $b_{i}$) were used to extrapolate the MicroHDI from 1991 to 2050, yearly.

### Projecting GHG emissions

We used CO_2_eq to associate the mass of gases emitted into one single variable $(e_{i,t} )$ (265 for N_2_O and 28 for CH_4_ [29]) from 2000 to 2010. Each emission grid was overlapped into the *microregional* geographic grid in order to reveal each *microregion’s* total emissions. When the emission grid was overlapped into one or more *microregions*, its proportional value was attributed to each *microregion*. Then, the total emission $e_{i,t}$ (in tons) was divided by the *microregion* area (in km^2^) in order to generate the emission intensity variable $\hat{e}_{i,t}$. Instead of emissions by population, the emission intensity variable represents emission patterns geographically associated to that *microregion*. By adopting this approach, we ensure that emissions are consistent with our emissions reduction’s proposal (described in the following sections), which is also geographically distributed.

The next step was to verify the existence of a correlation pattern between $\hat{e}_{i,t}$ and $d_{i,t}$, in the years for which observed data are available for both (2000 and 2010). We used linear regression ${\text{ln}}(\hat{e}_{i,t} ) = h_{t} d_{i,t} + g_{t}$ $(t = 2000,\;2010)$ to assess the correlation.

Once the correlation has been checked and found satisfactory, we projected future emissions based on the DAU approach, similarly as performed by Costa and co-workers [12]. Eq. 3 shows the regression model used to estimate the emission density for each *microregion*, where $\tilde{e}_{i,t}$ represents the metric of emission density, which is presented in tons CO_2_eq (km^2^)^−1^ in the *microregion i* in year *t* $(t = 2000,\;...,\;2010)$; $\tilde{d}_{i,t}$ is the estimated MicroHDI assessed by Eq. 2.

3 $${\tilde{e}_{i,t} = e}^{{h_{i} \tilde{d}_{i,t} + g_{i} }}$$

The *h*_*i*_ and *g*_*i*_ coefficients for each *microregion* were estimated by Nonlinear Ordinary Least Squares regression. In order to credit robustness to the set of estimated emissions, we selected as samples only the estimates for the *microregions* whose regressions in Eq. 3 had both coefficients statistically significant at 10% or less and R^2^ > 0.9. From these coefficients and owning $\tilde{d}_{i,t}$ for the years between 1991 and 2050, we projected the CO_2_eq emissions of each *microregion* for the same period. The total CO_2_eq released by *microregion i* at time *t* was assessed as *microregion’s* emission intensity times its area.

### Allocating emission reduction among Brazilian *microregions*

Assuming a proactive position from Brazil regarding international mitigation targets and SDG 13—Climate Action, this study designs and describes a reduction framework that distributes total national GHG emissions across *microregions* according to its human development level, which intrinsically accounts for SDGs 1, 3, 4 and 8—No Poverty, Good Health and Well-Being, Quality Education and Decent Work and Economic Growth, respectively. We used IPCC’s global scenarios [1] and Brazilian NDC [25] to determine some Brazilian commitments to reduce emissions (next section describe these commitments).

Legislative, socio-economic and structural adjustments may be required to enable such reductions, and that may include productive rearrangements, technological improvements, and changes in habits and social customs [37, 38]. From this observation, the period from 2011 until 2020 was determined as the *adjustment phase*, when it is expected that *microregions* adapt to less environmentally aggressive activities, since they will already be aware of their commitment to mitigate emission as from 2020. This period is also defined because the most updated data on MHDI is available for 2010.

After 2020, *microregions* with MicroHDI of at least 0.8 are required to reduce emissions proportionally to their human development level (Eqs. 4 and 5). The 0.8 threshold represents a very high *microregional* human development level, similarly to the MHDI and the HDI methodology. As a *microregion* becomes very highly developed, we assume that its responsibility over the country’s mitigation targets may be higher than the *microregions* below the 0.8 threshold. These *microregions* find themselves in the named *Responsibility domain* (see Fig. 5), in which they should be reducing their annual GHG emissions rates. Our framework to allocate emission reduction takes the *Responsibility domain* as the ideal development path for developed *microregions* and assures that all of them will achieve this path. By doing this, we provide more ground for developing *microregions* to freely decide over the activities required to achieve higher MicroHDI and facilitate their capacity to adapt to climate change, as recommended by some studies [14, 16–18]. Developing *microregions* (below 0.8 threshold) which are also reducing their annual GHG emissions rates find themselves in the *Best-case domain* (see Fig. 5). Developing *microregions* that are not reducing their annual GHG emissions rates find themselves in the *Fairness domain* (see Fig. 5). Reserving a *Fairness domain* for developing *microregions* may guarantee their development without the obligation to mitigate and without jeopardizing the national commitments. Finally, developed *microregions* that are not reducing their annual GHG emission rates find themselves in the *No-go domain* (see Fig. 5). This domain must be avoided because the resulting emissions would be inconsistent with any national climate mitigation goal in a region with already reasonably high human development.

Equations 4 and 5 operate the framework to allocate the emission reduction, where the *microregion i* reduces its emissions in year *t* according to the reduction rate $r_{i,t}$, which leads to the emission $\overline{e}_{i,t}^{total}$ lower than the expected emissions $\tilde{e}^{total}_{i,t}$. The *f* term is the proportionality constant $(f > 0)$ that may be changeable in order to determine the required reductions to ensure the achievement of the national mitigation goals

4 $$r_{i,t} = f(\tilde{d}_{i,t} - {0}{\text{.8)}}\quad \forall \,\,\tilde{d}_{i,t} \ge {0}{\text{.8}}\;and\;t \ge {2020}$$

5 $$\overline{e}^{total}_{i,t} = ({1} - r_{i,t} )\,\tilde{e}^{total}_{i,t} \quad \forall \,\,\tilde{d}_{i,t} \ge {0}{\text{.8}}\;and\;t \ge {2020}{\text{.}}$$

## Results and discussion

### MicroHDI and GHG emissions: observed trends

The average MicroHDI for Brazil (weighted by microregions’ population) was 0.408 in 1991, 0.543 in 2000 and 0.673 in 2010. Whereas the increase between 1991 and 2000 was 33%, the increase between 2000 and 2010 was 24%, reinforcing the assumption of sigmoidal shape of the MicroHDI development. The fastest development trend occurs in *microregions* of the Northeast region, historically the least developed region in Brazil, which is in line with recent developmental policies observed in this region. On the other hand, the slower development occurs in *microregions* in the South and Southeast regions, which are more developed.

**Fig. 1:**
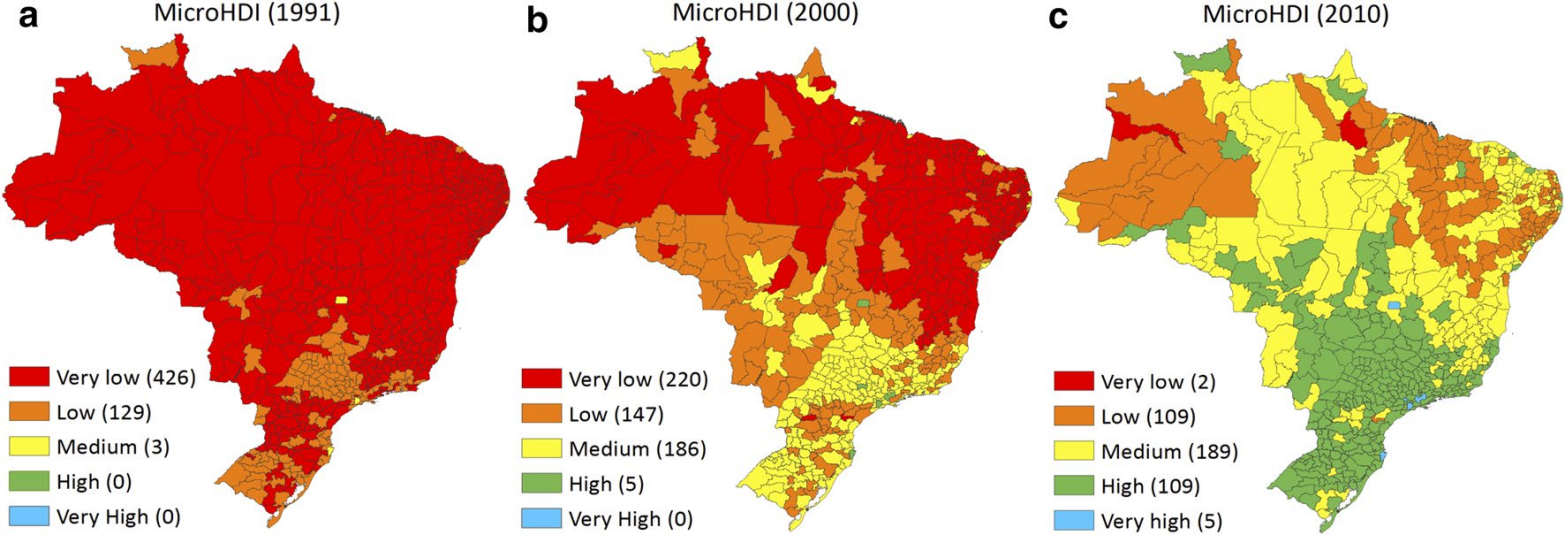
Brazilian *Microregional* Human Development Index (MicroHDI) from five human development ranges. MicroHDI values from Eq. 1, 1991 (**a**), 2000 (**b**) and 2010 (**c**)

Figure 1 shows the spatial distribution of MicroHDI in Brazil. We defined the levels of development as follows: Very low (MicroHDI < 0.5), low (0.5 ≤ MicroHDI < 0.6), medium (0.6 ≤ MicroHDI < 0.7), high (0.7 ≤ MicroHDI < 0.8) and very high (MicroHDI ≥ 0.8) [39, 40]. In 1991 (Fig. 1a) and 2000 (Fig. 1b) the very low development *microregions* were predominant; in 2010 (Fig. 1c) high development *microregions* appeared more often. Despite the advances, some regional differences remain, which becomes clearer by the constant discrepancy between the North-Northeast regions and the rest of the country.

**Fig. 2:**
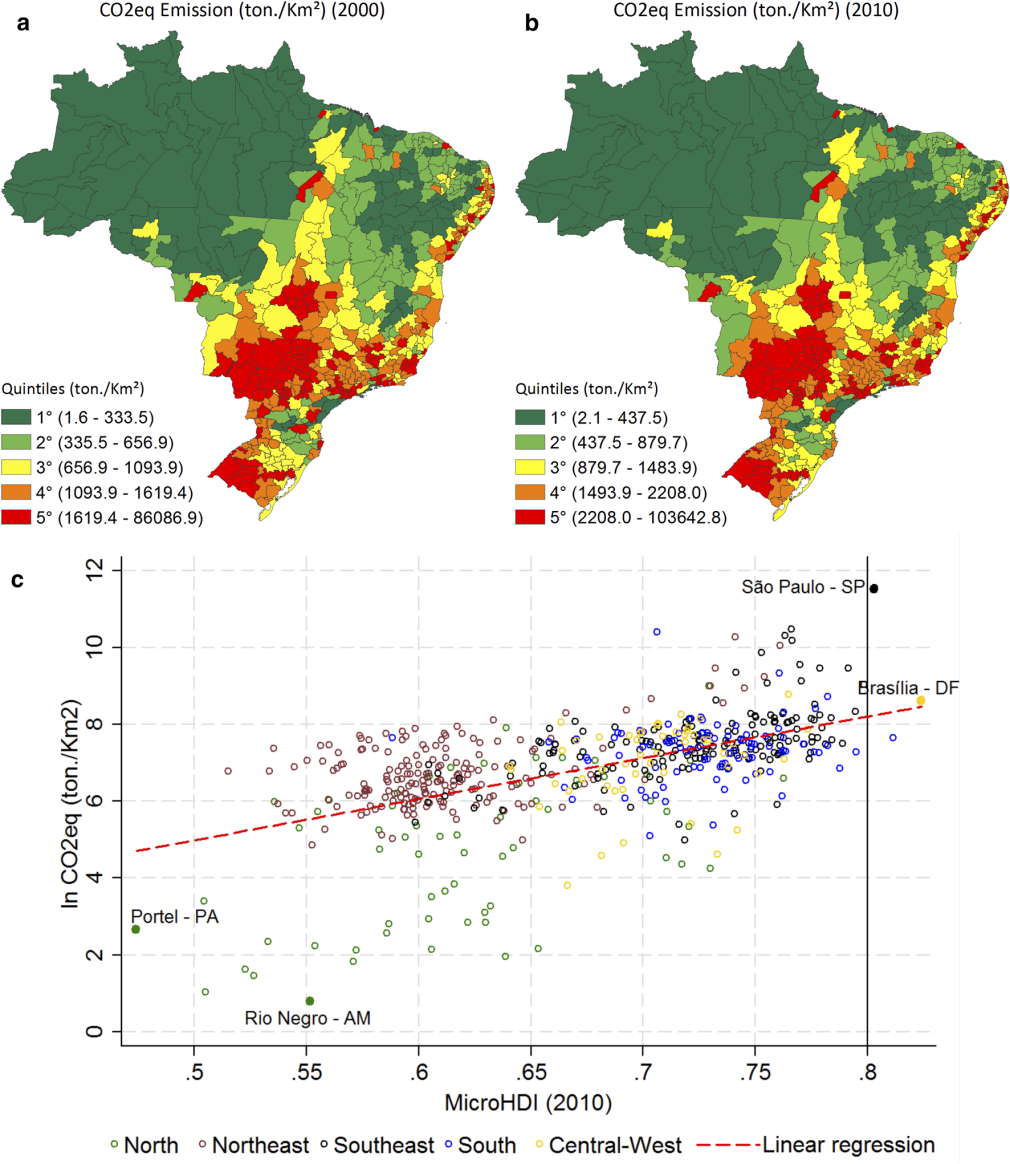
CO_2_eq emissions trends and their relationship with MicroHDI for Brazil. CO_2_eq emissions (ton/km^2^) in 2000 (**a**). CO_2_eq emissions (ton/km^2^) in 2010 (**b**). Correlation between MicroHDI and CO_2_eq emissions in 2010 (**c**). The red dashed line represents a least square fit through all values. The correlation coefficient is 0.55. The vertical dashed line represents the MicroHDI value of 0.8 representative of a very high development standard

According to EDGAR v4.2 FT2010 [28], the average CO_2_eq emissions in 2000 were about 1600 tons (km^2^)^−1^, while in 2010 this was 2077 tons (km^2^)^−1^, which accounts for a 30% increase. The Northeast and the South regions have increased emissions by approximately 33%, while the North region increased by 24%. Figure 2 shows the quantiles of emissions by area. The largest CO_2_eq emitters are located close to the state capitals and in the states Mato Grosso and Rio Grande do Sul, which are, together with Central West, a very important agriculture frontier in Brazil.

Furthermore, EDGAR shows that most of the emissions from state capitals are based on fossil fuel combustion [28], which is closely correlated with human development at global level [12] and with our fossil fuel sectorial results. In fact, when comparing Figs. 1c and 2b, it is remarkable that the highest MicroHDI regions are the largest CO_2_eq emitters (considering all sectors). The correlation between MicroHDI and lnCO_2_eq was 0.55 in 2000 and 2010. We present the result for 2010 in Fig. 2c. Despite the fact that the correlation values were not so high, there seems not to be a variation in the association of the variables of interest, which supports the DAU approach, and the assumption of future maintenance of the observed association as a key feature. The adoption of emission density (emission by area) is supported by the higher correlation between MicroHDI and GHG emissions when emissions are normalized by area than when they are normalized by population or without normalization. Our decision for considering various gases and sources made it possible to capture Brazilian heterogeneities, however it may decrease the potential correlations.

Most of the Northeast *microregions* are above the regression line (Fig. 2c) suggesting that the level of emissions, given its level of development, is above average. However, the Northeast region aggregates the least developed Brazilian economies. Considering CBDR, it may be understood that the Northeast temporarily postpones their mitigation efforts as leverage to restructure their socio-economic activity to higher standards. North *microregions* are largely below the regression line (Fig. 2c), suggesting a low level of emissions given their human development level. However, both conclusions should be considered in the context of not taking into account emissions from deforestation, since extensive agriculture expansion and extractive activity are relevant for some of those *microregions* [41, 42].

### Projecting MicroHDI

From the *microregions* in Eq. 2, we estimated the evolution of MicroHDI between 1991 and 2050. The model’s average adjustment (R^2^) for all the regressions was 0.99. The regressions presented better fitting in the Northeast region, showing significant $a_{i}$ and $b_{i}$ coefficients in all regressions. Nonetheless, in the Southeast *microregions*, only 85% of regressions were significant. Considering 558 regressions, 93% presented both coefficients significant at a 10% level.

**Fig. 3:**
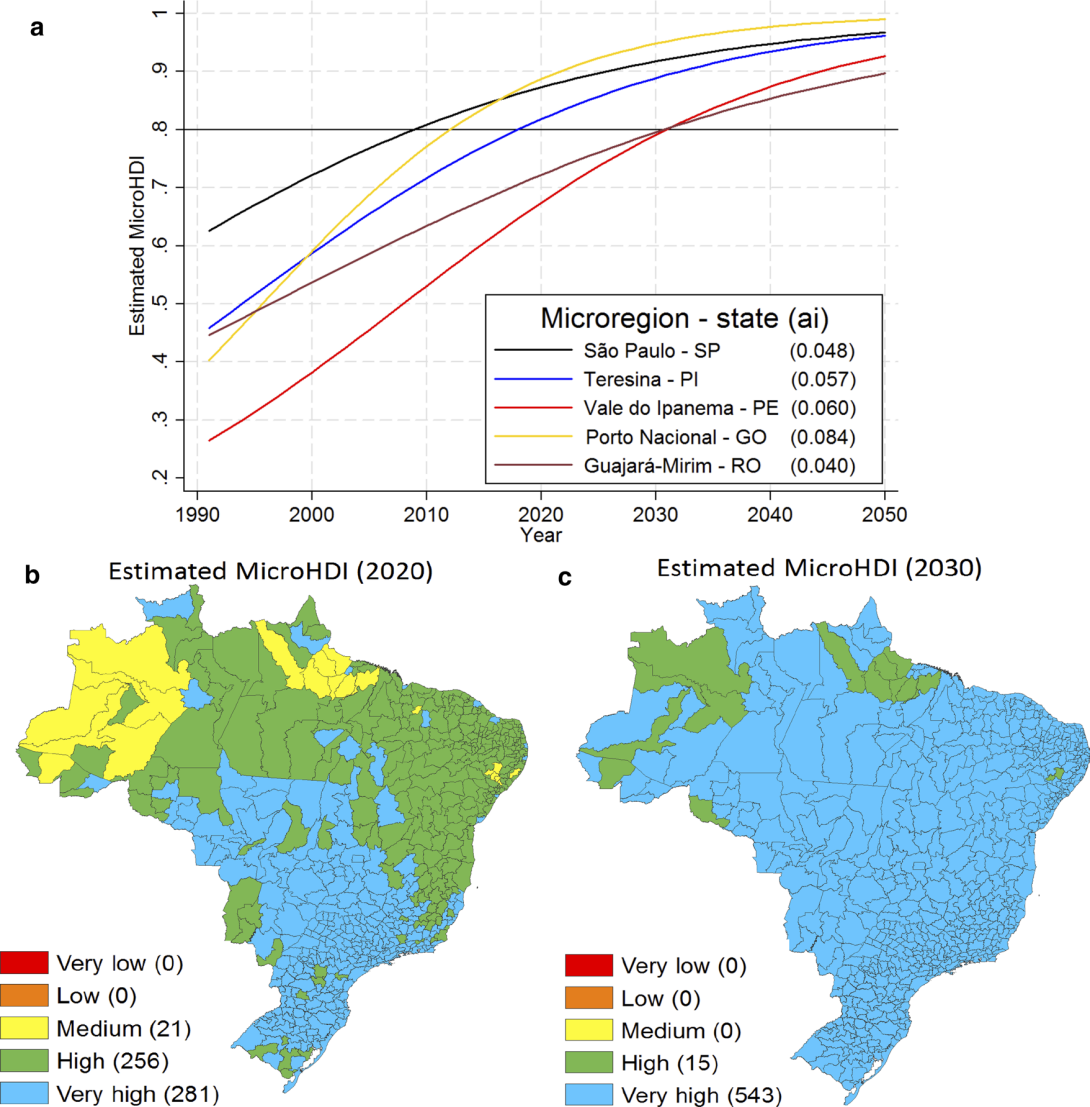
Projected MicroHDI for Brazil. Projected MicroHDI in a temporal perspective for some contrasting *microregions* (**a**). The horizontal line represents the MicroHDI value of 0.8, representative of a very high development standard, whereas a_i_ values in parentheses represent how fast a *microregion* develops. MicroHDI projected values from Eq. 2, 2020 (**b**). MicroHDI projected values from Eq. 2, 2030 (**c**)

Figure 3a shows the MicroHDI projections from a temporal perspective for some *microregions* of interest. The Porto Nacional *microregion* (Tocantins state) presented the fastest development ($a_{i}$ = 0.084). It achieved the very high development level (MicroHDI = 0.8) in 2013, and together with Brasília, is expected to be the first *microregion* to achieve the MicroHDI = 0.9 by the year 2022. It is well known that agribusiness and the industry are the core economic strategies of the development of this *microregion* [43]. However, such economic growth is associated with high environmental demands in the region, and may be unsustainable in the long run [43]. On the other hand, the *microregion* of Guajará-Mirin, a very poor location in the Brazilian North region (Rondônia state), presented the slowest development ($a_{i}$ = 0.040) in the sample and may achieve the very high development level (MicroHDI = 0.8) only by 2031. In general, Rondônia state is one of the deforestation leaders in Brazil [44], but apparently has not translated its resource depletion into regional development.

Figure 3b and c present future MicroHDI development from a geographical perspective for 2020 and 2030, respectively. Accordingly, the Brazilian development is expected to continue being characterized by the separation between the North-Northeast regions and the rest of the country by 2020 (Fig. 3b). Despite the discrepancies in time dependence of human development, all the *microregions* may reach a high development level by 2030 (MicroHDI = 0.7) (Fig. 3c) and very high after 2036 (MicroHDI = 0.8), which may be interpreted as reasonable progress towards SDGs 1, 3, 4 and 8. The estimations may be influenced by the period of the data used (1991–2010), during which several Brazilian indicators grew above average. But on the other side, given the current slowdown in the economic growth [45], some environmental and structural dilemmas in Brazil [46] and the recent global recession from 2020, our result may be understood as one of the several potential scenarios of socio-economic development.

Increases in living standards are commonly associated with increased environmental degradation [6]. This includes, for example, increased demand for prepared food products, such as meat and milk [47, 48], and the need of focusing on sustainable intensification of production as a way to increase yields in unproductive areas [49, 50]. Furthermore, increased life quality also includes a higher demand for high value-added durable goods such as private transportation and household appliances. This leads to increased energy demand and to higher GHG emissions, production costs and prices. It is reasonable to infer that if improvements in life quality do not come with productive rearrangements able to meet the new demands, gains in human development can be reflected as undesirable environmental contexts.

Therefore, the government and society have to guarantee that gains in social welfare do not negatively affect environmental benefits. In other words, it is important that the green economy ideas are followed, improving the population’s well-being while the environmental and ecological risks are minimized. According to UNEP [10], in a green economy, growth in income associated to the high level of human development “should be driven by public and private investments that reduce carbon emissions and pollution, enhance energy and resource efficiency, and prevent the loss of biodiversity and ecosystem services”. Public authorities have an essential role to play in this process, making public investments and carrying out political reforms.

### Projecting GHG emissions and the mitigation allocation framework

From the regressions made through Eq. 3, we estimated the CO_2_eq (km_2_)^−1^ emission by 2050 based on MicroHDI projected values $(\tilde{d}_{i,t} )$ (Table 1). Analyzing the Brazilian regions as the average result from its *microregions*, we see that the Southeast region presents high positive associations between human development and GHG emissions, insofar as a 0.1 $\tilde{d}_{i,t}$ increase is associated with approximately a 25.6% $\tilde{e}_{i,t}$ increase. In contrast, the North and Northeast regions have the lowest positive association among the variables, i.e., a 0.1 $\tilde{d}_{i,t}$ increase is associated with approximately a 21.5% $\tilde{e}_{i,t}$ increase.

**Table 1:** Estimated coefficients for Eq. 3 and projected variation in CO_2_eq (km^2^)^−1^ emissions $\left( {{\mathbf{\Delta \% }}{\varvec{e}}_{{\user2{i,t}}} } \right)$ related to 0.1 increase in MicroHDI by 2050

Coefficient	Brazil	North	Northeast	Southeast	South	Central west
$h_{i}$	5.74	4.40	5.64	5.95	5.84	6.33
$g_{i}$	2.08	1.95	1.95	2.26	2.09	2.01
$\Delta \% e_{i,t} = \exp^{{(h_{i} /10)}}$	23.1%	21.5%	21.5%	25.6%	23.2%	22.5%
_R2_	0.998	0.997	0.998	0.998	0.998	0.998

The high association between the Southeast regions’ development and emissions may indicate its association with high demography density, energy demand, and other factors that induce emissions too, resulting in a higher GHG emissions intensity. On the other hand, the country’s less developed regions (North and Northeast) present gains in human development related to low rates of GHG emissions in comparison with the most developed region (the Southeast). This might be related to mechanisms of technology transfer, governmental policies and a local economy not based on activities intensive in emissions. In general, the main lines of development seem to be maintained in Brazil as a whole, i.e. using fossil fuels and extensive agriculture. In addition, the big economic centers keep improving their own attractiveness to other people and industries, which generates sites even more emission-intensive.

Our results suggest that less developed Brazilian regions have not been basing their development in emission-intensive activities when compared to other Brazilian regions; instead, the most developed regions have. This indicates that, at least to some extent, CBDR is ongoing among Brazilian regions. However, such a dynamic has to be enhanced even more so that the development gap between North-Northeast and the other regions (as shown in Fig. 1) closes as soon as possible. Our methodological approach does not allow us to conclude either whether both the North and Northeast regions could take advantage of a more positive association between MicroHDI and GHG emissions or if the Southeast region should use less of this correlation. In a global perspective, our results are in agreement with the findings that show that less developed countries have been emitting less GHG than the developed ones [12]. These results also demonstrate that the sub-national scale application of the CBDR is comparable to the international one, which enhances the need for broadening such studies to other developing countries.

According to the EDGAR database, the analyzed *microregions* emitted 5.33 Gt CO_2_eq altogether in 2005 and 5.77 Gt CO_2_eq in 2010 [28]. In our estimation from Eq. 3, we calculated that the *microregions* emitted 5.13 Gt CO_2_eq in 2005 and 5.80 Gt CO_2_eq in 2010. The values from EDGAR v4.2 FT2010 [28] represent the basis for determining the mitigation needed to achieve the goals established by IPCC and NDC. IPCC provides probability scenarios of global temperature increase in 2100 given global emission in 2050 related to emission in 2010 [1]. We used three mitigation goals from IPCC, goal *a*, goal *b* and goal *c*, all of them having 2050 as the target year to achieve mitigation. Brazilian NDC [25] presents commitments to reduce GHG emissions in 2025 and 2030 according to emissions in 2005. We took two mitigation goals from Brazilian NDC, namely NDC25 and NDC30, having 2025 and 2030 as target years to achieve mitigation, respectively. Then we used each mitigation goal together with Eqs. 4 and 5 to determine the proportionality constant *f* required to achieve the mitigation targets. In Table 2 we provide the full description of the mitigation goals as well as the *f* term required to achieve the mitigation target in the referenced year.

**Table 2:** Characterization of emissions reduction goals and f term required to achieve mitigation targets

Mitigation target defined by:	*Goal a*	*Goal b*	*Goal c*	*NDC25*	*NDC30*
Reference year for mitigation	2010	2010	2010	2005	2005
Percentage mitigation related to the reference year	56.5%	26.50%	20%	37%	43%
Target year to achieve mitigation	2050	2050	2050	2025	2030
Probability of global increasing temperature in 2100 exceeds 2 ºC	24.5%	46.5%	69%	–	–
Target maximum GHG emission (Gt CO_2_eq)	2.51	4.24	4.62	3.32	3
Percentage mitigation related to expected emissions in 2020^a^	64.7%	40.4%	35.1%	53.3%	57.8%
Time available to achieve mitigation target from 2020 (years)	30	30	30	5	10
*f* term required to achieve mitigation target	4.7	3.6	3.3	7.6	10.6

^a^Our projections under the DAU approach estimated that Brazil may emit 7.12 Gt CO_2_eq in 2020 and we used this value as a basis to evaluate the necessary effort to achieve the target emission in each case (as we consider that mitigation efforts initiate only after the *adjustment phase*)

At the global scale, a reduction of 56.5% in CO_2_eq emissions in 2050 related to 2010 is required to keep at 24.5% the probability of increasing global temperature at 2ºC in 2100 (*goal a*). Similarly, a reduction of 26.5% results in the probability of temperature increase of 46.5% (*goal b*) and reducing emissions by 20% results in the probability of temperature increase of 69% (*goal c*). The NDC states GHG mitigation of 37% (*NDC25*) and 43% (*NDC30*) related to 2005, but does not relate it to any probability of global increasing temperature. Higher mitigation will always relate to lower probabilities of future global warming. In contrast, if there is no mitigation, the probability of global temperature increasing in 2100 exceeds 2ºC is 100% [1].

As a preliminary analysis, we evaluated the time available to operate the mitigation according to the target years: 2025 for *NDC25*, 2030 for *NDC30* and 2050 for *goal a*, *goal b* and *goal c*. Both NDC’s goals are short term (5 and 10 years) but their mitigation commitments are high (37% and 43%), as well as their mitigation related to expected emissions in 2020 (53.3% and 57.8%). It makes NDC’s commitments more audacious than IPCC’s, which is reflected as high *f* values (7.6 and 10.6). Although we intended to include NDC’s goals in our analysis, we understand that its short time to mitigate and its high mitigation commitment are features that would impair its application. Data used for the estimates do not contemplate the period of recession that Brazil has been experiencing since 2015 nor the socioeconomic effects of COVID-19. This would lead to decreases in the growth rate, to less GHG emissions, and would make the NDC’s goals easier to achieve while impairing improvements in human development. From now on, we use only the IPCC’s goals to illustrate the application of the mitigation’s framework proposed because of their longer time for implementation.

Intended to make our analysis more robust, we also structured two alternative mitigation proposals: (i) MicroHDI threshold of 0.7 to start reductions and; (ii) reducing the *adjustment phase* to the period 2010–2015, with reductions starting in 2015 at 0.8 threshold. Using the 0.7 threshold, we observed a lower constant *f* necessary to achieve the pre-established reduction goals: *goal a*, *f* = 2.9; *goal b*, *f* = 2.2; *goal c*, *f* = 2.1. Although this represents smoother reduction pathways, we believe that the 0.7 threshold does not characterize a development level in which the *microregions* are sufficiently capable to start reductions without compromising their standards of living. Finally, we also considered the shorter *adjustment phase* with the reductions starting in 2015. This way, there would be no difference in constant *f* to achieve the 2050 goals, but some *microregions* would have to reduce emissions earlier. In general, taking into consideration some political and economic changes required in order to start reductions, the *adjustment phase* is inherent and shortening it would be an incentive for the *microregions* to deviate from the proposed framework. From this point on, we concluded that our main methodological approach with the MicroHDI threshold of 0.8 and the *adjustment phase* up to 2020 is the most functional for us to derive our mitigation allocation framework.

**Fig. 4:**
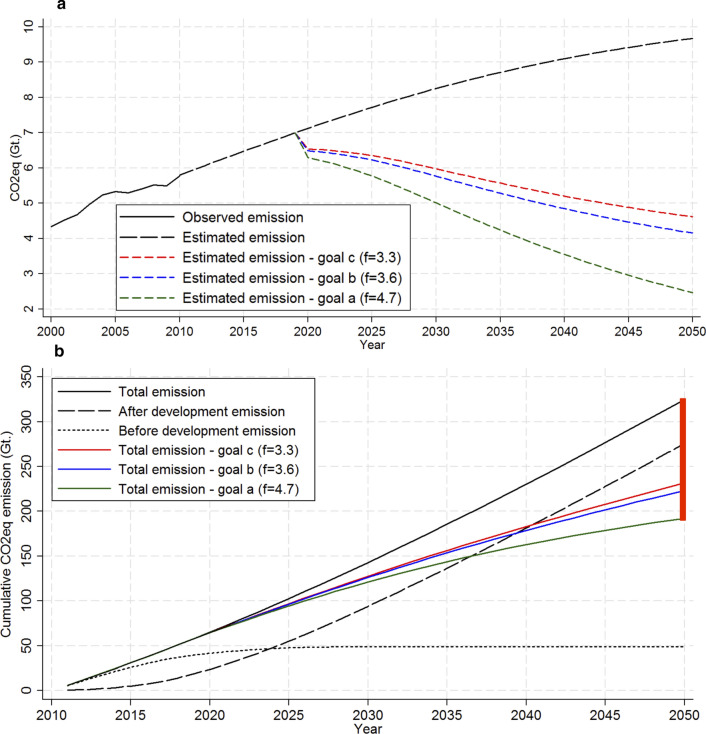
Projected and cumulative CO_2_eq emissions for a Development As Usual (DAU) approach according to three reduction goals for Brazil. Annual CO_2_eq emissions projection and the mitigation goals implications for Brazil (**a**). Cumulative CO_2_eq emissions from 2011 to 2050 (**b**). The red bar at the right edge of the frame illustrates the range of cumulative emissions reduction achievable under the proposed reduction *goal a* (~ 135 Gt CO_2_eq). The CO_2_eq mitigation goals indicate reductions in 2050 related to 2010. In the *goal a*: 56.5%; *goal b*: 26.5% and *goal c*: 20%

We further present the annual emissions at the country level in Fig. 4a. Accordingly, the estimated Brazilian total emissions in 2050 are 9.67 Gt CO_2_eq, which represents a 59% increase over the observed value in 2010. The proposed mitigation scenarios described how to achieve a reduction between 4.5 and 6.5 Gt CO_2_eq under the CBDR statements. If the *goal a* reduction was adopted, *goal c* would be achieved in 2033 and *goal b* in 2036.

In addition, Fig. 4b depicts the CO_2_eq accumulated in the atmosphere from 2011 until 2050. In 2011, the share of emissions related to the *microregions* whose MicroHDI is above 0.8 was near zero. In 2024, the emissions of developed *microregions* exceed the emissions of developing *microregions*. In 2036, all the regions are expected to be very highly developed and the accumulated emissions released before the development may stabilize around 50 Gt CO_2_eq. Between 2011 and 2050, Brazil would release about 325 Gt CO_2_eq in the atmosphere in the absence of any mitigation policy. Figure 4a and b demonstrate that if Brazilian emissions remain as in the past (DAU trajectory) the country is not progressing towards meeting SDG 13.

Regarding CBDR, the 50 Gt CO_2_eq emissions arising from less developed regions are justified as a necessary step towards development. In contrast, the 225 Gt CO_2_eq released by *microregions* after the development should be the focus of mitigation efforts. Our proposed framework demonstrates how to allocate 130 Gt CO_2_eq reduction (under *goal a*) in a way that only developed regions mitigate. Reducing this amount of emissions and not burdening less developed *microregions* is an advance towards both maintaining the global temperature as well as pursuing human development.

**Fig. 5:**
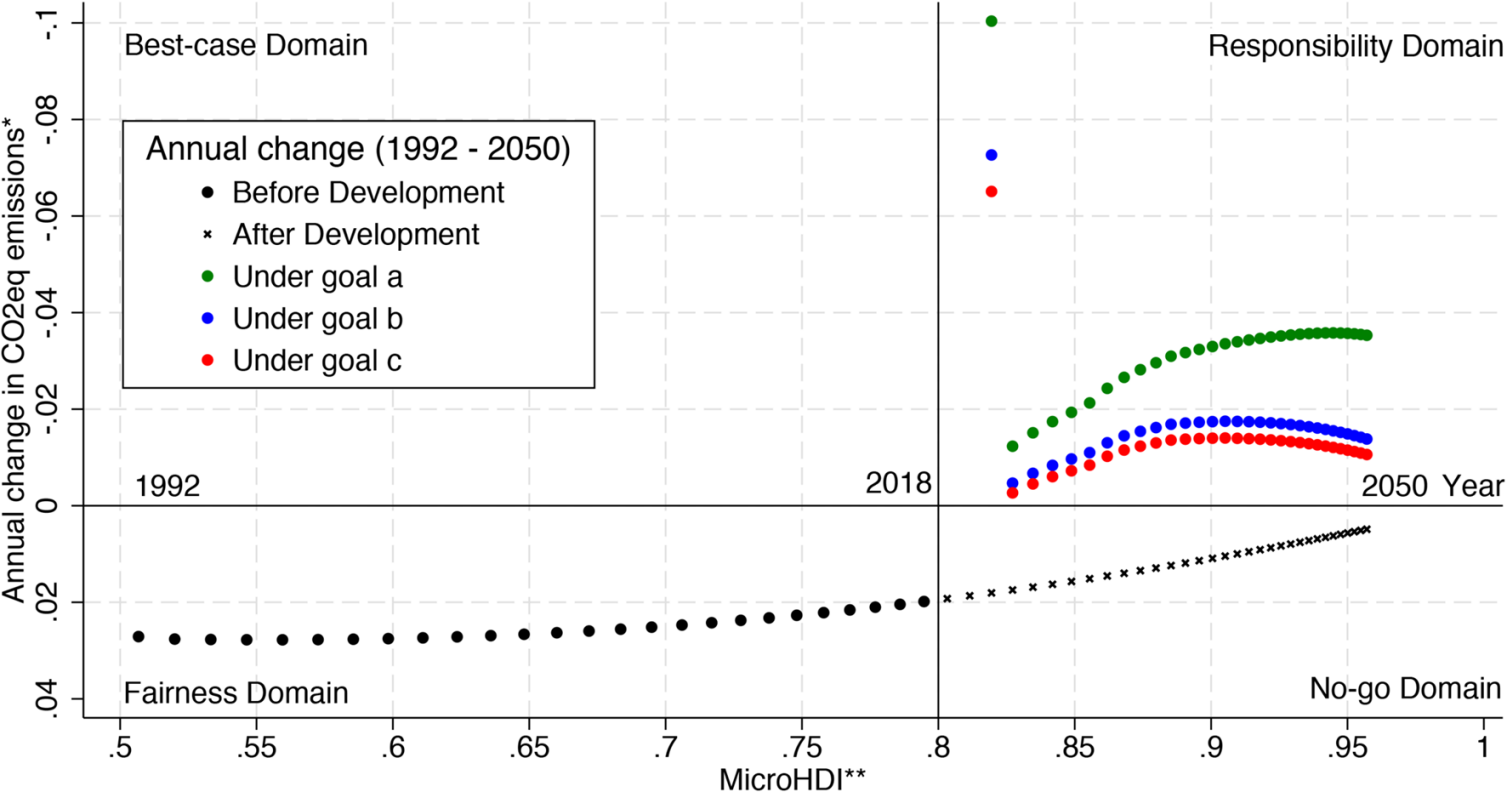
CO_2_eq emissions reduction framework based on MicroHDI for Brazil (1992–2050). MicroHDI in the figure represents an average of each *microregion* MicroHDI weighted by its population in 2010 and considering the 436 *microregions* in the sample. Note that the *y*-axis is inverted, similarly to Costa and co-workers [12]. The vertical line indicates the human development threshold of 0.8 MicroHDI, representative of very high development standards

Figure 5 presents the national annual variation in emissions (observed and projected) and considers the effect of the three mitigation goals in national GHG emissions. Considering Brazilian *microregions* as a whole, that is, the *microregional* results aggregated over the country and weighted by each *microregions’* population in 2010, we observe that Brazil has been following a trend toward the *No-go domain*. It highlights the importance of reduction efforts such as the one presented here for this specific country. According to our population weighted results, Brazil may be over the very high development threshold in 2018, and the emissions may still be in an increasing path. The colored dots in Fig. 5 elucidates the effect of our mitigation framework in taking Brazilian emission’s path into the *Responsibility Domain*. The initial-colored dots with high negative annual change in CO_2_eq emissions are the result of high human development regions getting into the reduction scheme and the adjustment phase should be used to soften this transition by adopting less emission intensive activities.

**Fig. 6:**
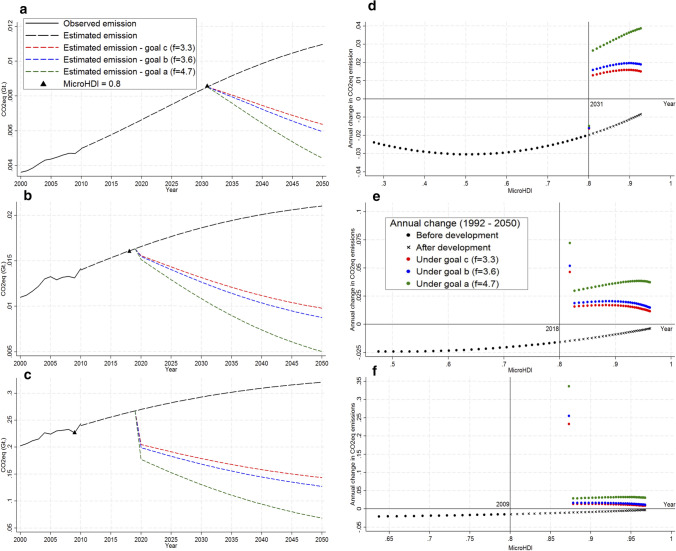
Annual CO_2_eq emissions (2000–2050) and CO_2_eq emissions reduction framework based on MicroHDI (1992–2050) for some contrasting Brazilian *microregions*. Annual CO_2_eq emissions observed and estimated and the mitigation goal implications for Vale do Ipanema—PE (**a**), Teresina—PI (**b**) and São Paulo—SP (**c**). Annual change in CO_2_eq emissions over the years under DAU approach and under the emissions reduction framework for Vale do Ipanema—PE (**d**), Teresina—PI (**e**) and São Paulo—SP (**f**). The y-axis of the Cartesian plane has been reversed in order to follow the same theoretical reasoning presented by Costa and co-workers [12]. The vertical solid line indicates the human development threshold of 0.8 MicroHDI, representative of a very high development standard. The CO_2_eq mitigation goals indicate reductions in 2050 related to 2010. In the *goal a*: 56.5%; *goal b*: 26.5% and *goal c*: 20%

Figure 6a–c shows the time emission projection and the implications of the mitigation goals in three *microregions* to illustrate distinct characteristics concerning MicroHDI evolution and GHG emissions. The development patterns of these *microregions* are heterogeneous and clear examples of how our framework accounts differently for each.

Furthermore, Fig. 6d–f shows the variation trend of emissions over the years under the DAU approach and under reduction schemes. Firstly, the *microregion* Vale do Ipanema (Fig. 6a and d) in the Northeast region is one of the least developed Brazilian *microregions*; it is expected to achieve the very high MicroHDI level in 2031, which implies the allowance of keeping a positive GHG emission growth rate until then because this *microregion* is currently in the *Fairness domain*. Secondly, Teresina (Fig. 6b and e), a medium developed Brazilian *microregion* in the Northeast, is expected to be developed in 2018 and it is supposed to initiate mitigations in 2020. Until 2018, this *microregion* is in the *Fairness domain*; from 2018 to 2020 (*adjustment phase*), it is in the *No-go domain*; and after 2020 the reduction scheme places it in the *Responsibility domain*. Finally, São Paulo (Fig. 6c and f), the country’s most developed *microregion*, became developed in 2009 and has been in the *No-go domain* since then. This *microregion* has been keeping high development levels without mitigating emissions; consequently, it must mitigate proportionally to its current very high development level just after the *adjustment phase* (2020).

**Fig. 7:**
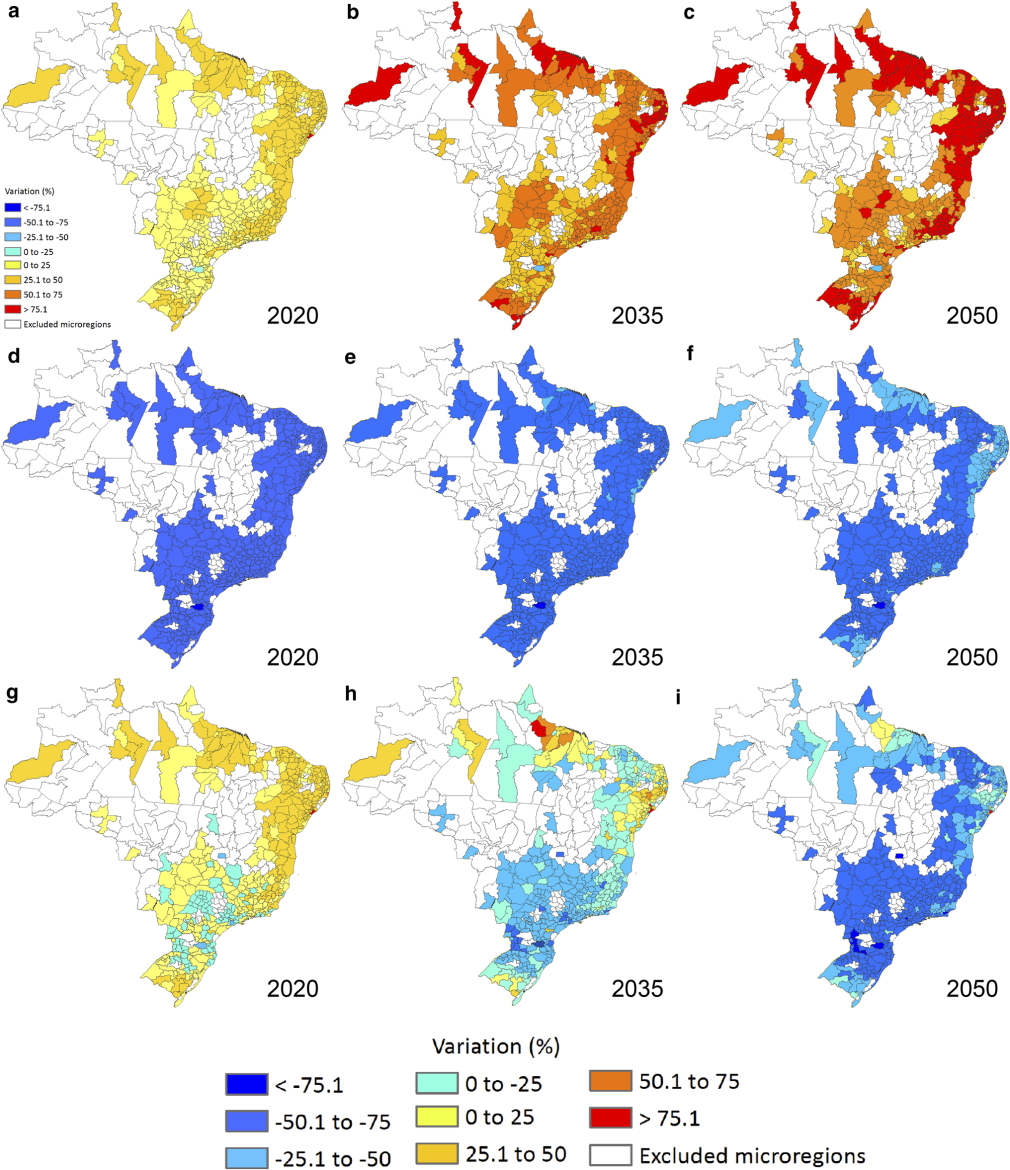
Percentage variation of CO_2_eq emissions related to estimated emissions in 2010 according to three different scenarios for Brazil. Percentage variation of CO_2_eq emissions according to DAU trajectory (**a**–**c**). CO_2_eq emissions reductions over DAU estimations considering a hypothetical linear reduction pathway, in which the term $r_{i,t}$ in Eq. 4 becomes a constant equal to 0.26 (without indexations) (**d**–**f**). Reduction pathways over DAU estimations according to our proposed framework (**g**–**i**). From **a**–**c**, there is no mitigation and, therefore, mitigation targets from Table 2 are not met. From **d**–**i**, mitigation burdens are set to achieve national mitigation *goal a* from Table 2. From **d**–**f**, mitigation burdens are evenly distributed while from **g**–**i** they are accounted for according to our framework. “Excluded *microregions*” indicate not statistically significant results

Figure 7 presents the percentage variation for the years 2020 (Fig. 7a–g), 2035 (Fig. 7b–h) and 2050 (Fig. 7c–i) related to the estimated emissions in 2010 according to three different scenarios: (i) DAU approach; (ii) a hypothetical linear reduction and; (iii) our mitigation allocation framework. In general, Fig. 7 presents how our mitigation allocation framework works geographically and differs from the other comparison scenarios.

In the DAU approach maps (Fig. 7a–c), emissions keep evolving as a function of MicroHDI, without mitigation and without accomplishing any of the hypothetical mitigation targets. We observe that *microregions* that already release high CO_2_eq will experience lower variations in GHG emission, while the ones with lower current emissions are expected to elevate their emissions by higher rates. Most of the *microregions* that will increase their emissions are located in the Northeast region.

In the hypothetical linear reduction proposal (Fig. 7d–f), the term $r_{i,t}$ from Eq. 4 becomes a constant equal to 0.26 (without indexations). With this change, Brazil will achieve the mitigation *goal a* without redistributing mitigations according to MicroHDI levels. We verified that the impact of reduction is, at most, between 50 and 75% as early as 2020 for all *microregions* considered. This is an unfair burden to the *microregions* below the very high development threshold and these mitigations could compromise their objectives of human development. In addition, new accomplishments in development (increasing MicroHDI) are not related to greater efforts to reduce GHG emissions (increasing $r_{i,t}$).

Considering our mitigation allocation framework from Eqs. 4 and 5 (Fig. 7g–i), we observe heterogeneities over the *microregions* and over the time being accounted as a more equally distributed mitigation burden. At first, we see *microregions* from Southeast, Central West and South having negative variations in emission, while *microregions* from North and Northeast will only have negative variations afterwards. In this sense, the more developed *microregions* will have to mitigate first, enabling the less developed ones to mitigate only after its development, without compromising the Brazilian mitigations target.

Overall, results demonstrate that Brazil is progressing towards higher socioeconomic standards (SDGs 1, 3, 4 and 8) but not regarding to climate change (SDG13) if MicroHDI and CO2 emissions evolve in the future as it was in recent years. Even though according to the CBDR principle it could be fair for a developing country like Brazil to base its development in emission intensive actives [12, 17], it is also expected that societies assume a proactive role regarding mitigation of climate change. Brazil has taken this active role by pledging robust emissions mitigation in its NDC, but the allocation of mitigation burdens and the effects of sub-national inequalities have been only minimally discussed [21]. Our allocation framework describes a fair distribution of mitigation burdens that ensure progress over all five SDGs discussed within this paper. The implementation of such sustainable pathway could occur by taking the principles of green economy into account, especially in the least developed regions.

## Limitations of our framework

Although theoretically consistent with previous work [12] and with CBDR, we recognize that our approach can only be regarded as an approximation exercise in some aspects. Despite the diversity of emission sources considered by the EDGAR database, it does not account for emissions from cutting down trees and logging. On the one hand, this can be a major constraint to the Brazilian context, as one of the main sources of emission reduction refers to the reduction of Amazon and Cerrado deforestation [30]. In contrast, some authors consider that historical deforestation data is still highly uncertain [44, 51–53], which could lead to less accurate estimates. Because mitigation in the land use change sector has been the most vigorous one in Brazil [30], our results are conservative estimations in terms of potential mitigation. Aspects like technological transfer and innovation, as well as regulatory frameworks, and the economic spillover effects of allocating higher burdens to the most developed regions cannot be anticipated and could benefit from further analysis in future work. In this paper focused on applying our emission reduction framework and other methods to better describe future emissions pathways and human development scenarios and the control of the realized mitigation are welcome and compatible with a green economy proposal. Finally, the assumption of one DUA scenario could benefit from an association with Shared Socioeconomic Pathways [54] to produce a series of baseline scenarios accounting for different storylines of wealth and population in Brazil.

## Final remarks

The transition to an economy with low-carbon and social inclusive pathways has specificities among regions around the globe because, according to UNEP, “it depends on the specifics of each country’s natural and human capital and on its relative level of development” [10]. Often, certain locations achieve a high level of human development at the expense of environment damages, such as high levels of GHG emissions. At the same time, it is necessary to consider that the concept of green economy implies associating environmental quality and “improved human well-being and social equity” [10]. Therefore, we seek to analyze the connections between green economy and climate mitigation by developing an analytical reference for policy makers on how to allocate mitigation burdens timely and geographically, based on regional human development. For this, we studied the Brazilian regions, given the heterogeneities of a country with continental dimensions.

Based on the CBDR principle, our model estimated 50 Gt of cumulative CO_2_eq emissions as a pre-condition for raising all Brazilian *microregions* to very high human development (MicroHDI ≥ 0.8) from 2011 to 2036. This represents less than one sixth of total Brazilian cumulative emissions in the DAU projection from 2011 to 2050 (325 Gt CO_2_eq). This way, we demonstrate how to disentangle human development in low developed regions from GHG mitigation burdens by allocating these burdens to regions at a very high human development, which are required to mitigate 190 Gt CO_2_eq if Brazil intends to meet *goal a*.

This dynamic crosstalk between GHG emissions reduction and development level is likely to encourage the adoption of policies that favor both issues. It is reasonable to assume that no society wants to go back in terms of standards of living, so in our simulations the governmental regulator would have to deal with both the increasing demand for higher standards of living and the increasing emission reduction rate. Political proposals based on dynamic frameworks are extremely important, as the imposition of this trade-off is an incentive to adopt sustainable development methods and to move forward to the *Responsibility domain*. The control of the realized level of reduction, however, depends largely on societal engagement for steering political decisions towards this implementation. In this sense, our work also contributes to raise awareness and to further discussions about more fair distributions of mitigation burdens in a green economy context.

Beyond Brazil, our framework is of global importance because we allocate high mitigation burdens to most developed Brazilian regions, which are the ones more able to design sustainable technological solutions and more economically relevant in the international context. By adopting this framework, the whole country would posit itself as an international reference for addressing both environmental and socioeconomic development, which is a broad development perspective emphasized by the Sustainable Development Goals.

## Supplementary Information

Additional file1 (CSV 963 KB)

Additional file2 (CSV 5 KB)

Additional file3 (DOCX 27 KB)

## Data Availability

The datasets generated during the current study are available from the corresponding author on reasonable request.
