# Supplementary material for: Human development, greenhouse gas emissions and sub-national mitigation burdens: a Brazilian perspective
Source: Discov Sustain. 2021 Aug 9;2(1):35. doi: 10.1007/s43621-021-00044-9 (PMC8350932; doi:10.1007/s43621-021-00044-9)
Supplement: Supplementary file 3 — Additional file3 (DOCX 27 KB) [file 43621_2021_44_MOESM3_ESM.docx]

**Supplementary Material**

**Municipal Human Development Index**

The MHDI is available at UNDP for the years 1991, 2000 and 2010 [31]. MHDI considers health, education and income dimensions. Each dimension has individual indexes that are then combined to formulate the MHDI. Eq 1-4 describes these dimensions and the methodology to calculate the MHDI.

The health index (*MHDI_H*) is the average number of years that a person born in a given municipality would live from birth keeping the same mortality patterns.

$MHDI\_H=\frac{\left(life expectance at birth-25\right)}{\left(85-25\right)}$ (1)

The education index (*MHDI_E*) is represented by (i) the education of the adult population as the percentage of people who are 18 years old or older with completed elementary school and (ii) the school flow of young people measured as the arithmetic mean of the percentage of children aged 5 to 6 attending school, the percentage of young people aged 11 to 13 attending the final years of elementary school, the percentage of young people aged 15 to 17 with completed elementary school, and the percentage of young people aged 18 to 20 with completed high school.

 (2)

The income index (*MHDI_I*) is built by the per capita municipal income (reference values of 2010). The sum of income of all residents is divided by the number of people who live in the city including children and people with no income record.

(3)

The three indexes are then combined as follows to compose the MHDI.
